# Dr. David Hamilton

**Published:** 1909-11

**Authors:** 


### OBITUARY

DR. DAVID HAMILTON, of Batavia, died at the Batavia Hospital, October 5, 1909, aged 70 years. He was born in Kingston, Ont., and was the son of John M. Hamilton, a Canadian statesman.

Dr. Hamilton graduated at Queen's University, Kingston, April 26, 1862. He saw service in the British and American armies. Dr. Hamilton came to Batavia about 33 years ago and had practised his profession there ever since until his last brief illness. He was a well bred gentleman and an excellent physician.

